# Species Differences in Platelet Protease-Activated Receptors

**DOI:** 10.3390/ijms24098298

**Published:** 2023-05-05

**Authors:** Stephanie A. Renna, Steven E. McKenzie, James V. Michael

**Affiliations:** Department of Medicine, The Cardeza Foundation for Hematologic Research, Thomas Jefferson University, Philadelphia, PA 19107, USA

**Keywords:** antiplatelet agents, BMS-986120, protease-activated receptor 4 (PAR4)

## Abstract

Protease-activated receptors (PARs) are a class of integral membrane proteins that are cleaved by a variety of proteases, most notably thrombin, to reveal a tethered ligand and promote activation. PARs are critical mediators of platelet function in hemostasis and thrombosis, and therefore are attractive targets for anti-platelet therapies. Animal models studying platelet PAR physiology have relied heavily on genetically modified mouse strains, which have provided ample insight but have some inherent limitations. The current review aims to summarize the notable PAR expression and functional differences between the mouse and human, in addition to highlighting some recently developed tools to further study human physiology in mouse models.

## 1. Introduction

Platelets are small, discoid, anucleate cells derived from megakaryocytes [1,2]. At 1–3 μm in diameter, they are the smallest cellular component of blood and have an average lifespan of 7–10 days before being cleared from circulation [3,4,5]. The most well-known role of platelets is to maintain hemostasis. When the vascular endothelium is compromised, nearby platelets immediately begin to accumulate at the injury site and form a tight plug that prevents further bleeding [6,7]. Over time, the clot grows and is stabilized by a network of polymerized fibrin. An appropriate hemostatic response is critical to balance between continuous bleeding (hemorrhage) and vessel occlusion (thrombosis). The aim of antiplatelet drugs is to reduce platelet activity, thus preventing dangerous blood clots.

Human platelets express a repertoire of surface receptors that are important for platelet activation and thus provide potential targets for antiplatelet drugs. These include G protein-coupled receptors (GPCRs), such as protease-activated receptors (PARs) 1 and 4, the purinergic receptors P2Y1 and P2Y12, and a thromboxane A2 (TxA2) receptor known as TP receptor [8,9]. GPCRs have seven transmembrane domains and act through heterotrimeric G-proteins to trigger specific downstream responses [10,11]. GPCRs are common targets of anti-platelet drugs. Dual antiplatelet therapy (DAPT) is widely used in the prevention of thrombotic events [12,13,14,15]. DAPT traditionally consists of the cyclooxygenase-1 (COX-1) inhibitor aspirin, which prevents arachidonic acid metabolism and subsequent TxA2 generation, and a P2Y12 inhibitor, such as clopidogrel or ticagrelor [16,17,18]. More recently, there has been increasing enthusiasm for anti-platelet drugs targeting PARs, as the human platelet PAR system offers a unique opportunity to more finely modulate the balance of hemostasis and thrombosis due to the biphasic activation and differing thrombin affinities of PAR1 and PAR4 [19]. PAR1 inhibition using vorapaxar has shown limited efficacy in the prevention of major thrombotic events, but seems to protect against dangerous limb ischemia [20]. The PAR4 inhibitors BMS-986120 and BMS-986141, which have a wider therapeutic window than conventional anti-platelet agents, have shown considerable promise in preventing thrombotic events [21,22]. 

Mouse models are widely used in the platelet field, despite considerable species differences as shown by multiple studies [23]. One major difference is in their platelet PAR expression profiles, namely that (1) unlike human platelets, mouse platelets express Par3 and Par4, and (2) human and mouse PAR4 are functionally different from one another. With the recent focus on PAR4 (including newly developed inhibitors) in the hemostatic response as well as under thrombotic conditions, the inherent difficulty of studying PAR receptor biology *in vivo* is more crucial than ever. In this review, we will explore signaling, development of inhibitors, species divergence, and newly developed tools to study PAR4 biology.

## 2. Platelet Protease-Activated Receptors

As their names suggest, PAR1 and PAR4 are activated by proteases, with the primary activator of both receptors being thrombin, though each are activated by additional proteases [24,25,26]. Proteolytic cleavage of the N-terminus of PARs reveals a tethered ligand sequence that interacts with the ligand binding site to cause activation. They each signal through Gq and G12/13 to activate phospholipase Cβ (PLCβ) and RhoA, respectively, with the major outcomes being granule secretion, integrin activation, and shape change [8]. 

The first known PAR, PAR1, was cloned in 1991 by Vu et al. [27]. By this time, thrombin’s role as a potent platelet agonist was well known, but the mechanism by which a protease acts on platelets was still poorly understood [28]. In this study and in a follow-up study, Vu et al. described a novel mechanism by which thrombin, a serine protease, interacts with PAR1 and cleaves its N-terminus, revealing a tethered ligand [29]. In the decade following the discovery of PAR1, three more PARs were discovered, PAR4 being the only other PAR expressed on human platelets [24,30]. Due to its higher affinity for thrombin, PAR1 was initially thought to be the more attractive target for anti-platelet drug development. However, when vorapaxar, the first PAR1 antagonist to receive U.S. Food & Drug Administration (FDA) approval, was found to cause bleeding complications in patients with peripheral artery disease [31], the focus began to shift to PAR4.

### 2.1. Protease-Activated Receptor 4

**Figure 1:**
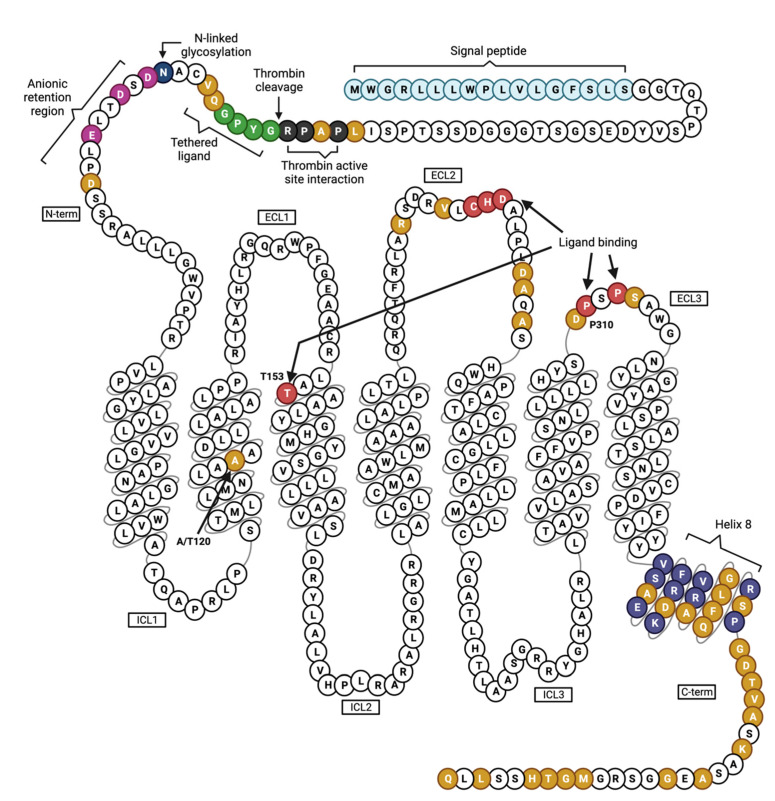
Human PAR4 and its putative functional domains. PAR4 is a 7 transmembrane GPCR. The N-terminus contains a signal peptide (light blue), thrombin active site interaction region (dark gray), tethered ligand (green), anionic retention region (magenta), and one N-linked glycosylation site (dark blue). Thr153 in transmembrane (TM) 3 and several residues in the second and third extracellular loops (ECLs) are critical for ligand binding (red). The common variant p.Ala120Thr is located in TM2. The C-terminus contains helix 8 (purple). Residues in yellow differ between human and mouse PAR4. Figure was created using Biorender.com.

Like all other PARs, PAR4 activation is initiated by proteolytic cleavage of the N-terminus [27,32]. This cleavage occurs between Arg57 and Gly58 and leads to the exposure of the tethered ligand sequence GYPGQV. There is a CHD binding motif in the extracellular loop (ECL) 2 that is believed to be required for tethered ligand binding in PAR4 and all other PARs as well [33,34,35]. An elegant structural study by Han et al. in 2020 used histidine hydrogen/deuterium exchange to identify additional critical players in the ligand binding site (LBS); notably, transmembrane regions (TM) 3 and 7 exhibited considerably lower deuteration upon receptor activation by thrombin, suggesting that these TMs are critical for formation of the binding pocket. Computational modeling predicted a likely interaction between Gly47 of the tethered ligand and Thr153 in TM3, further supporting TM3’s role in the LBS. Moreover, ECL3 was identified as a “gatekeeper” of the LBS, as it appeared to prevent the tethered ligand from interacting with the LBS prior to cleavage [36]. A snake plot depicting the sequence of human PAR4, highlighting its functional domains and variation in amino acids compared to mouse Par4, is illustrated in Figure 1.

PAR4 is activated by other proteases in addition to thrombin, including trypsin, plasmin, and the neutrophil-derived protease cathepsin G (CTSG) [37,38]. While most structure/function studies to date have focused on thrombin activation, one recent study demonstrated that CTSG cleaves PAR4 at a site distinct from thrombin, Ser67, revealing an alternate tethered ligand that activates the receptor in a manner similar to thrombin. They showed that a monoclonal antibody raised against the thrombin cleavage site failed to inhibit platelet aggregation induced by CTSG. Moreover, the 11mer sequence they identified as an alternative tethered ligand (RALLLGWVPTR) was able to induce full aggregation as well as αIIbβ3 activation and granule secretion [39]. The physiological significance of this remains to be elucidated.

Aside from proteases, PAR4 can be activated experimentally using a selective synthetic activating peptide, AYPGKF-NH2 (also referred to as PAR4-AP). A structural study by de la Fuente et al. demonstrated by histidine hydrogen/deuterium exchange that activation of PAR4 by thrombin induces a conformational change resulting in a “protected” C-terminus, but this conformational change was not observed upon stimulation with PAR4-AP [35]. The implications of this finding are presently unclear, but it strongly suggests that activation with PAR4-AP is not a true recapitulation of PAR4 activation by thrombin or potentially other proteases. Upon activation with a protease or activating peptide, PAR4 signals through heterotrimeric G proteins Gq and G12/13, which likely interact with intracellular loop (ICL) 2 [40], to induce granule secretion, integrin activation, and shape change.

### 2.2. PAR Regulation

Due to the irreversible nature of PAR activation, receptor trafficking is critical for signal regulation. Following activation and signal transduction, GPCRs traditionally undergo rapid serine/threonine phosphorylation of the cytoplasmic tail by GPCR kinases (GRKs). Phosphorylation then results in β-arrestin binding, G protein uncoupling, and ultimately receptor internalization and degradation [41,42]. PAR4, however, likely does not conform to this exact model of negative regulation [43].

The cytoplasmic tail of PAR4 contains a total of eight serine and threonine residues that are putative targets for phosphorylation by GRKs. In comparison, the PAR1 C-terminus contains 15 serine and threonine residues. PAR1 desensitization in platelets has been extensively studied; upon agonist stimulation, the carboxy terminus is rapidly phosphorylated by GRK5/6 which leads to the recruitment of β-arrestin 1 and decoupling of G proteins [44,45,46]. PAR4 desensitization, on the other hand, has not been studied as thoroughly. A recent report has demonstrated calcium mobilization after activation of human PAR4 was not affected by genetic deletion of GRK6, the predominant GRK expressed in human platelets, in Meg-01 cells [45]. An earlier study showed that PAR4 was not phosphorylated at all in a transfected fibroblast system and that mutation of the eight serine and threonine residues had no effect on PAR4 signaling [47]. The apparent lack of phosphorylation suggests that PAR4 is desensitized and internalized much more slowly than PAR1 [43], which may play a role in its sustained calcium signaling and contribution to generation of procoagulant platelets.

Unlike PAR1, internalization of PAR4 is not very well understood. One study by Smith et al. in 2016 describes a potential mechanism of PAR4 endocytosis; as is also the case with PAR1, PAR4 undergoes clathrin-mediated endocytosis after activation. In classical clathrin-mediated endocytosis, adaptor protein-2 (AP-2) recognizes phosphorylated residues in the C-terminus of GPCRs and facilitates binding of other clathrin adapter proteins such as β-arrestin [48]. Like PAR1 [49], PAR4 internalization may also occur in a β-arrestin-independent manner, relying on AP-2, clathrin, and dynamin. In contrast to PAR1, however, deletion of most of the C-terminus of PAR4 had no effect on receptor internalization, suggesting that AP-2 interacts with a different domain of PAR4. As is the case with other PARs, the irreversible nature of PAR4 activation results in the internalized receptor being trafficked through the early endosome to the lysosome for degradation [50]. Sometime during the internalization process, β-arrestin is recruited to the PAR4 signaling complex, potentially with the help of P2Y12, and facilitates activation of the mitogen-activated protein kinase (MAPK) pathway, leading to sustained Akt activation [50].

### 2.3. PAR Antagonists

Thrombin is the most potent platelet agonist, and inhibiting its effects specifically on platelets to prevent thrombosis while preserving hemostasis is an attractive idea. In 2014, the FDA approved the drug vorapaxar, a first-in-class PAR1 inhibitor, for use in patients with peripheral artery disease (PAD) or a history of myocardial infarction (MI). Vorapaxar is highly selective and effectively irreversible, interacting with the tethered ligand binding region of the extracellular face of the receptor to prevent activation [51]. Phase 3 trials determined that PAR1 inhibition is a viable approach to the prevention of ischemic events, but in some cases at the expense of increased bleeding risk, particularly in patients with a history of stroke [52,53]. A phase 4 trial recently demonstrated that addition of vorapaxar to mono- or dual-antiplatelet therapy had no effect on clot characteristics or coagulation in patients with a history of MI or PAD, though it may have anti-inflammatory effects [54]. A more recent study examined endothelial cell biomarkers in a follow-up to the TRACER trial [52] and reported increases in pro-inflammatory biomarkers such as angiopoietin-like 4 (ANGPTL4) in patients who received vorapaxar treatment, suggesting that vorapaxar causes endothelial dysfunction [55]. 

Considering the increased bleeding risk and limited efficacy associated with PAR1 inhibition, PAR4 antagonism has come to the forefront. The novel small molecule BMS-986120 was developed by Bristol-Myers Squibb and its efficacy was demonstrated in a cynomolgus monkey model of occlusive arterial thrombosis [56]. Compared to the P2Y12 antagonist clopidogrel, BMS-986120 exhibited a wider therapeutic window in thrombosis and bleeding models. These findings were followed up with human *ex vivo* studies in the Prospective Randomized Open-Label Blinded End Point (PROBE) phase 1 clinical trial. BMS-986120 was shown to selectively inhibit PAR4-AP-induced aggregation, P-selectin expression, and platelet-monocyte aggregates. Additionally, whole blood assays showed that it significantly reduced thrombus size at high, but not low, shear rates in a Badimon perfusion chamber [21]. In the single-ascending-dose (SAD) and multiple-ascending-dose (MAD) phase 1 trials, it was demonstrated that BMS-986120 works equally well against the common, hyperreactive PAR4 variant Thr120 [22]. Moreover, the alternative lead compound, BMS-986141, has shown similar efficacy with minimal bleeding and is currently recruiting for a phase 2a trial for coronary artery disease (NCT05093790) [57]. These results suggest that PAR4 is a promising target for antiplatelet therapeutics. PAR4 is additionally attractive as an antiplatelet target as its expression is relatively constrained; however, inflammatory stimuli have been shown to cause upregulation of PAR4 in various tissues and cell types [58,59,60,61]. This will need to be taken into consideration for all future studies of PAR4 antagonism.

## 3. Species Differences and Implications

### 3.1. PAR Landscape of Mouse and Human Platelets

**Figure 2:**
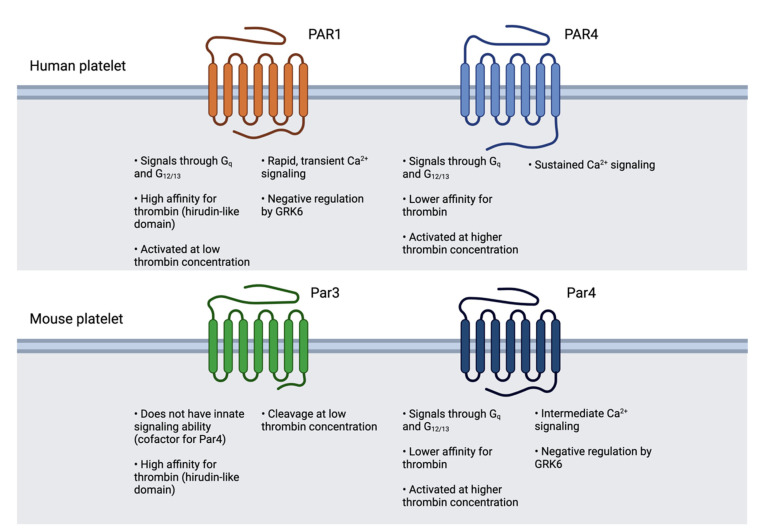
Human and mouse platelet PAR landscapes. Human and mouse platelets express different sets of PARs. Human and mouse platelets express different sets of PARs. Human platelets express PAR1 and PAR4, both of which signal through Gq and G12/13. Mouse platelets express Par3 and Par4, and of the two only Par4 is capable of signaling; Par3 acts as a cofactor for Par4. These differences pose a challenge when studying platelet PARs *in vivo*. Figure was created using Biorender.com.

Both human and murine platelets express two thrombin receptors. Human platelets express PAR1 and PAR4, both of which are responsive to thrombin and other proteases and ultimately trigger calcium flux, though with disparate kinetics [9]. PAR1 contains a hirudin-like domain which binds thrombin with high affinity, facilitating activation of PAR1 at thrombin concentrations as low as 0.5 nM [29]. PAR4 lacks this domain and instead contains an anionic “retention region” that allows it to interact with thrombin only at a higher concentration, around 0.8–1 nM [32,62]. Mouse platelets, on the other hand, express Par3 and Par4, and only Par4 has signaling capability. In response to sub-nanomolar concentrations of thrombin, Par3 interacts with thrombin via its hirudin-like domain and brings it into proximity with Par4, facilitating its cleavage and activation [63]. A summary of the platelet PAR landscape of human and mouse platelets is depicted in Figure 2. 

### 3.2. Human vs. Mouse PAR4

Since its discovery, several studies have been published on the role of PAR4 in hemostasis, thrombosis, and inflammatory processes in mice [64,65,66]. One major caveat is that mouse platelets express Par3 as the second thrombin receptor, rather than Par1. Multiple groups have attempted to achieve PAR1 expression in mouse platelets with no success (discussed in more detail below) [67,68]. In addition, while human and mouse platelets both express PAR4, their sequences are considerably variable in important functional domains (Table 1). Such differences pose a significant challenge when trying to extrapolate information about how human PAR4 functions *in vivo*.

#### 3.2.1. Activation

The tethered ligand sequences differ between the two receptors at positions 5 and 6, human PAR4 being GYPGQV and mouse Par4 being GYPGKF. Notably, the widely used PAR4-AP, or AYPGKF-NH2, more closely aligns with the mouse Par4 tethered ligand. In fact, it has been shown that the peptide GYPGQV-NH2 is approximately 50% as potent compared to GYPGKF-NH2 in human platelet aggregation [9]. This suggests that there is a difference in how each tethered ligand interacts with the ligand binding domain to cause activation. While it is not fully understood how tethered ligands activate PARs, it has been demonstrated that the extracellular loop 2 (ECL2) is critical for determining the specificity of the PAR receptor for its ligand [76]. 

There remains an open question of whether the neutrophil-derived protease CTSG can activate mouse platelets via mouse Par4, and thus whether such an interaction can be explored *in vivo*. Multiple studies have demonstrated that CTSG isolated from human neutrophils activates human platelets via PAR4 but fails to activate mouse platelets [37,77,78]. It was previously thought that CTSG cleaves human PAR4 at the thrombin cleavage site [37], but mouse Par4 contains the same cleavage site and yet is not activated by CTSG. In support of the alternative CTSG cleavage site proposed by Stoller et al. [39], mouse Par4 does not contain this sequence, which may explain why it is not activated. At least one *in vivo* study has implicated that mouse CTSG can activate mouse Par4 [79]. However, human and mouse CTSG are evolutionarily distinct proteases. CTSG gained tryptic function in addition to its original chymotryptic function during primate evolution, and exhibits reduced cleavage efficiency compared to its earlier mammalian ancestor [80].

#### 3.2.2. Signaling and Desensitization

In 2014, Edelstein et al. first described a single nucleotide variant (SNV) in transmembrane helix (TM) 2 of human PAR4, p.Ala120Thr, which had a significant effect on PAR4 activation by PAR4-AP [69]. The EC50 of thrombin is significantly lower for the Thr120 variant, resulting in enhanced G protein signaling, calcium mobilization, and ERK phosphorylation [70,71,72,73]. For mouse Par4, the residue at this position is valine (Val132). The well-characterized and robust differences in human PAR4 signaling resulting from a single amino acid change in TM2 is highly suggestive of additional signaling differences between human and mouse PAR4.

Many GPCRs including protease-activated receptors have an amphipathic helical motif that connects TM7 to the cytoplasmic tail, referred to as helix 8. This eighth helix often contains at least one cysteine residue that is dynamically acylated, allowing the cytoplasmic tail to be tethered in close proximity to the plasma membrane [74]. This post-translational modification is thought to modulate GPCR signaling, desensitization, and internalization. Helix 8 has low sequence homology between human and mouse PAR4, which likely results in additional signaling differences between the two. 

**Figure 3:**
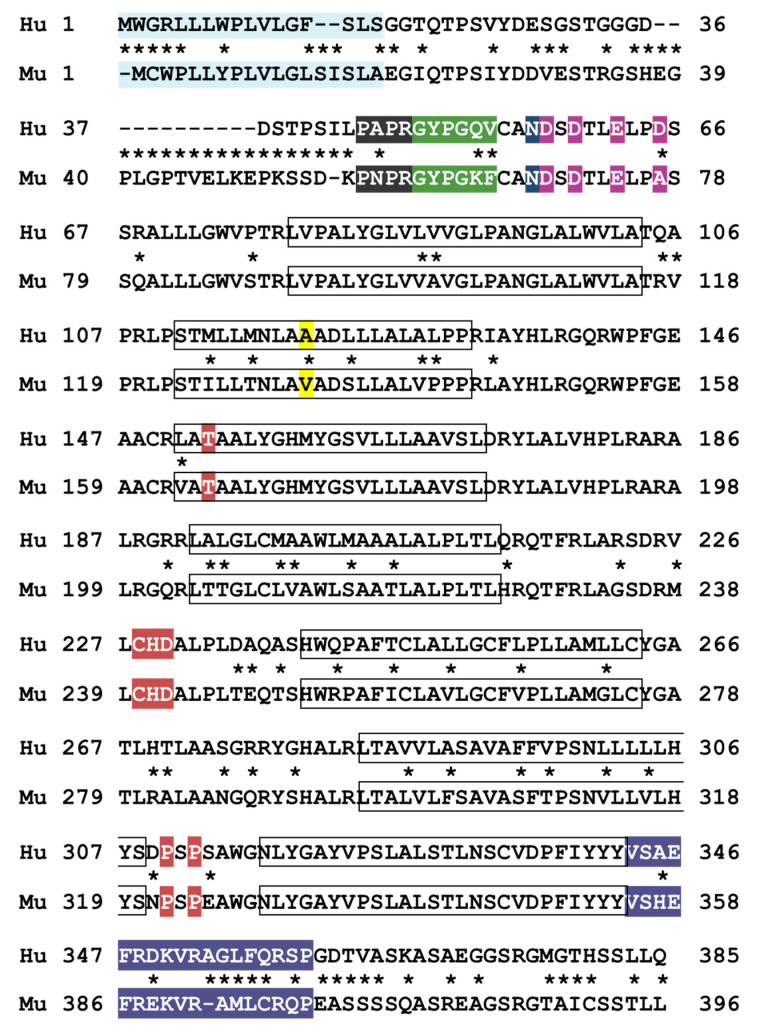
Sequence alignment of human and mouse PAR4 protein. The following functional domains are highlighted: signal peptide (blue), thrombin active site interaction region (gray), tethered ligand (green), N-linked glycosylation site (dark blue), anionic retention region (magenta), ligand binding site (red), and helix 8 (purple). The common variant p.Ala120Thr is in yellow. The 7 transmembrane domains are in boxes. Asterisks denote sequence differences.

The cytoplasmic tails of GPCRs generally contain multiple serine/threonine residues which are rapidly phosphorylated by GRKs after activation to promote decoupling of G proteins and contribute to receptor desensitization [75]. The cytoplasmic tail of human PAR4 contains six serine and two threonine residues while mouse Par4 contains two additional serine residues. Unlike PAR1, which is rapidly phosphorylated after activation by thrombin, PAR4 is apparently not phosphorylated at all [47]. Moreover, Chen et al. demonstrated that GRK6 phosphorylates the cytoplasmic tails of human PAR1 and mouse Par4, but not human PAR4, which is highly suggestive of differential negative regulation between the two receptors [45]. A sequence alignment of human and mouse PAR4 highlighting areas of high and low homology is depicted in Figure 3.

### 3.3. Other Species

Mice are among the most common model organisms used for platelet studies, due to the ease of generating transgenic models or genetic deletions, in addition to convenience and cost. However, it should be noted that it is not the only model organism available. A list of common species used in translational research and their platelet PAR expression can be found in Table 2. Of the commonly used small laboratory animal species, guinea pig platelets are most aligned with human platelets, as they express both PAR1 and PAR4, but they also express PAR3, which complicates interpretation of the thrombin response [81]. Additionally, guinea pig platelets are considerably less sensitive to stimulation with the PAR1 activating peptide SFLLRN, suggesting major differences between human and guinea pig PAR1 [81]. Rodent models in general also pose a challenge for studying PAR antagonists. Multiple PAR1 antagonists, including vorapaxar, have been shown to be ineffective at blocking rat and mouse PAR1 activation [51,81]. The binding affinity of the PAR4 antagonist BMS-986120 is high to monkey PAR4, but has no appreciable binding to rat or guinea pig PAR4. Furthermore, BMS-986120 has a weak affinity to mouse PAR4, indicating mouse studies using this inhibitor may be challenging [56]. 

Regardless of the animal model system used, it is critical to note that transgenic, knockout, or differential expression of platelet PARs may yield unanticipated results due to any number of alterations in the proteome and/or interactions in downstream signaling and regulation. It is crucial that in interpreting data from animal models, may or likely will not completely recapitulate the human response. However, studies that provide detailed functional characterization from well-designed and controlled experiments are a vital step in further understanding human physiology

## 4. Humanized Mouse Model of Platelet PARs

### 4.1. Human PAR1 Mice

Ideally, there would be a mouse model expressing both human PAR1 and PAR4. Despite multiple attempts, the humanized platelet PAR1 mouse model remains elusive. In 2014, Arachiche et al. reported their attempt to generate such mice by using the platelet-specific promoter for mouse GPIbɑ to drive either mouse or human PAR1 expression on platelets. While they detected successful insertion of their cDNA constructs, in both cases the platelets remained insensitive to PAR1 activating peptide (PAR1-AP) SFLLRN in a platelet aggregation assay. They again attempted to express human PAR1, this time under the human integrin ɑIIbβ3 promoter, but the platelets remained insensitive to PAR1 activation [67]. 

A second attempt was reported in 2016, where French et al. aimed to knock human PAR1 into the mouse Par3 locus. Similar to the 2014 study, the authors confirmed PAR1 expression at the DNA level, but platelets from PAR1 knockin mice failed to aggregate in response to PAR1-AP. In fact, when stimulated with thrombin, the platelets responded as Par3 KO platelets would, further indicating that they had successfully targeted mouse Par3. Additionally, the authors did not detect PAR1 protein within or on the surface of PAR1 knockin platelets, suggesting a failure of either transcription or translation of human PAR1 mRNA or protein. This was the last peer-reviewed attempt at generating platelet PAR1 mice [68].

### 4.2. A Novel Human PAR4 Transgenic Mouse Model

In 2022, our group reported novel human PAR4 transgenic mice and compared their platelets to wild type (WT) platelets, which was the first time any study has directly compared human and mouse PAR4 functionality in an otherwise equivalent platelet environment [82]. A human PAR4 genomic clone bearing the Ala120 variant was introduced into the mouse genome via pronuclear injection, resulting in mice expressing both human and mouse Par4. Transgenic founder mice were subsequently outcrossed with Par4 KO mice, achieving mice that express only human PAR4, denoted as PAR4 tg/KO. The initial report characterizing these mice demonstrated that isolated platelets from human PAR4 transgenic mice were more responsive to activation with PAR4-AP when assessed for a multitude of platelet activity markers including integrin activation, granule secretion, sustained intracellular calcium, etc. Regulation of PAR4 by G protein-coupled receptor kinase 6 (GRK6) was directly addressed in humanized mice, demonstrating that mouse, but not human, PAR4 is regulated by GRK6 and that regulation mechanisms are conserved in the newly developed mouse model [45]. 

The interest in generating humanized PAR4 mice has been pursued by other groups simultaneously. Denorme, Bray, Campbell, and colleagues have also recently generated humanized PAR4 mice expressing either the Ala or Thr120 SNV using CRISPR/Cas9 with a specific insertion to replace mouse F2rl3. Abstracts providing preliminary findings have detailed SNV-dependent differences in stroke severity and neutrophil extracellular trap (NET) formation [83,84]. The PAR4 inhibitor BMS-986120 significantly attenuated disease markers following the same stroke model, implicating a critical involvement of PAR4 signaling in brain injury.

## 5. Conclusions

Mono- or dual-antiplatelet therapy is a critical pharmacological intervention for a variety of cardiovascular diseases (CVD), in addition to inherited or acquired blood clotting disorders. Due to the prevalence of these disorders worldwide, there has been a continuous effort to improve the tools and/or find the optimal DAPT for any given disease. The potent activation of platelets via thrombin is well established, which has facilitated a massive effort to find potent and specific therapeutics to reduce PAR signaling. In recent years, PAR4 has become a target of interest for anti-platelet therapies. Emerging evidence suggests the elimination of the prolonged signaling of thrombin by targeting PAR4 provides protection in inflammation and thrombotic events, while maintaining the critical hemostatic response of PAR1. To date, mouse models pose both a significant opportunity and challenge for studying PAR biology both *ex vivo* and *in vivo* that directly translates to human physiology, largely because they do not express the same set of PARs as human platelets. With the generation and characterization of humanized PAR4 mice, we are one step closer to the necessary tools needed in pursuit of understanding human PAR4 function *in vivo*.

## Figures and Tables

**Table 1:** Domain differences between human and mouse PAR4 and their potential consequences.

Domain	Potential Consequences	References
Tethered ligand	Differences in tethered ligand sequence may impact receptor activation.	[9]
Ligand binding site	Differences in extracellular loops (ECL) 2 and 3 may affect the strength and/or kinetics of G protein signaling.	[33,34,35]
Transmembrane helix 2	Single nucleotide difference at human PAR4 position 120 may affect the strength and/or kinetics of G protein signaling.	[69,70,71,72,73]
C-terminus	(a) Differences in helix 8, particularly the lack of cysteine palmitoylation in human PAR4, may affect signaling, desensitization, and/or internalization. (b) Differences in the cytoplasmic tail may affect GRK phosphorylation and negative regulation.	[45,47,74,75]

**Table 2:** Platelet PAR expression across different species.

Species	Platelet PAR Expression
Human	PAR1, PAR4
Mouse	PAR3, PAR4
Rat	PAR3, PAR4
Rabbit	PAR3, PAR4
Guinea pig	PAR1, PAR3, PAR4
Monkey	PAR1, PAR4

## Data Availability

Not applicable.
